# Conductometric Immunosensor for *Escherichia coli* O157:H7 Detection Based on Polyaniline/Zinc Oxide (PANI/ZnO) Nanocomposite

**DOI:** 10.3390/polym13193288

**Published:** 2021-09-26

**Authors:** Sawsan Mutlaq, Borhan Albiss, Anas A. Al-Nabulsi, Ziad W. Jaradat, Amin N. Olaimat, Mohammad S. Khalifeh, Tareq Osaili, Mutamed M. Ayyash, Richard A. Holley

**Affiliations:** 1 Department of Nutrition and Food Technology, Jordan University of Science and Technology, P.O. Box 3030, Irbid 22110, Jordan; shmutlaq17@agr.just.edu.jo (S.M.); anas_nabulsi@just.edu.jo (A.A.A.-N.); tosaili@just.edu.jo (T.O.); 2 Nanomaterials Laboratory, Department of Applied Physics, Jordan University of Science and Technology, P.O. Box 3030, Irbid 22110, Jordan; 3 Department of Biotechnology and Genetic Engineering, Jordan University of Science and Technology, P.O. Box 3030, Irbid 22110, Jordan; jaradatz@just.edu.jo; 4 Department of Clinical Nutrition and Dietetics, Faculty of Applied Medical Sciences, The Hashemite University, P.O. Box 330127, Zarqa 13133, Jordan; aminolaimat@hu.edu.jo; 5 Department of Basic Medical Veterinary Sciences, Jordan University of Science and Technology, P.O. Box 3030, Irbid 22110, Jordan; mskn@just.edu.jo; 6 Department of Clinical Nutrition and Dietetics, College of Health Sciences, University of Sharjah, Sharjah P.O. Box 27272, United Arab Emirates; 7 Department of Food Science, College of Agriculture and Veterinary Medicine, United Arab Emirates University (UAEU), Al Ain P.O. Box 15551, United Arab Emirates; mutamed.ayyash@uaeu.ac.ae; 8 Department of Food and Human Nutritional Sciences, University of Manitoba, Winnipeg, MB R3T 2N2, Canada; rick.holley@umanitoba.ca

**Keywords:** *E. coli* O157:H7, polyaniline, zinc oxide nanoparticles, immunosensor, antibody

## Abstract

A conductometric immunosensor was developed for the detection of one of the most common foodborne pathogens, *Escherichia coli* O157:H7 (*E. coli* O157:H7), by conductometric sensing. The sensor was built based on a polyaniline/zinc oxide (PANI/ZnO) nanocomposite film spin-coated on a gold electrode. Then, it was modified with a monoclonal anti-*E. coli* O157:H7 antibody as a biorecognition element. The fabricated nanostructured sensor was able to quantify the pathogens under optimal detection conditions, within 30 min, and showed a good detection range from 10^1^ to 10^4^ CFU/mL for *E. coli* O157:H7 and a minimum detection limit of 4.8 CFU/mL in 0.1% peptone water. The sensor efficiency for detecting bacteria in food matrices was tested in ultra-heat-treated (UHT) skim milk. *E. coli* O157:H7 was detected at concentrations of 10^1^ to 10^4^ CFU/mL with a minimum detection limit of 13.9 CFU/mL. The novel sensor was simple, fast, highly sensitive with excellent specificity, and it had the potential for rapid sample processing. Moreover, this unique technique for bacterial detection could be applicable for food safety and quality control in the food sector as it offers highly reliable results and is able to quantify the target bacterium.

## 1. Introduction

Food safety has become a major concern worldwide due to the increased incidence of illnesses related to the consumption of food products contaminated with pathogens. The World Health Organization has reported that these illnesses affect 1:10 people worldwide, causing almost 420,000 deaths each year [1]. *Escherichia coli* O157:H7 is one of the most common foodborne bacterial pathogens and it belongs to the enterohemorrhagic *E. coli* (EHEC) group [2,3]. This bacterium is mainly found in the gut of cattle as its main reservoir [4,5]. Although the incidence of foodborne illness associated with *E. coli* O157:H7 is low compared to other pathogenic bacteria, it can cause severe symptoms and life-threatening illnesses such as hemorrhagic colitis (HC) and hemolytic uremic syndrome (HUS), which can lead to kidney failure if not treated [4,6]. Therefore, it is urgent that an effective and rapid technique be developed to detect *E. coli* O157:H7 in food products throughout the production and distribution chain.

Alongside conventional detection techniques, several culture-independent techniques have evolved and been modified to facilitate the detection of *E*. *coli* O157:H7 [7]. For instance, *E. coli* O157:H7 has been detected by amplifying specific genes using PCR, application of enzyme-linked immunosorbent assays (ELISA), or optical or mass-sensitive biosensors [8,9,10]. However, these methods require trained personnel, expensive materials and equipment, extended periods are often needed to obtain reliable results, sometimes they cannot be used directly on-site, and they may involve the use of toxic substances. Therefore, the development of sensitive, specific, cost-effective, and less laborious detection techniques for *E. coli* O157:H7 is needed.

Recently, a number of electrochemical biosensors have been described for the detection of *E*. *coli* O157:H7, which have high specificity, a reasonable detection limit, low cost, portability, near-immediate detectability, simplicity, and have the capacity for modification with nanomaterials [11,12,13]. Conducting polymers such as polyaniline (PANI) have been extensively applied in electrochemical sensors due to their electro-activity (conductivity), which can enhance the sensitivity of the sensor. Furthermore, PANI is highly compatible with biological molecules, eco-friendly and easy to prepare [14,15]. The doping of metal oxides such as gold (Au), silver (Ag), titanium (TiO_2_), zinc oxide (ZnO) and carbon nanostructures in PANI can enhance the electrical affinity, electrical conductivity and lower the ionization potential of the composite [16]. Chowdhury et al. [17] proposed a label-free Au/PANI impedimetric sensor for the detection of *E*. *coli* O157:H7 using the antibody-antigen binding method. However, the sensor exhibited a detection limit of 10^2^ CFU/mL. In addition, the preparation of PANI nanocomposites with other metal derivative nanostructures such as zinc oxide, carbon dots or magnetic nanoparticles represent a new route for enhancing PANI performance by creating new materials with complementary or synergistic properties between PANI and the metal oxide [18]. Pangajam, Theyagarajan and Dinakaran [12] developed an electrochemical sensor for the detection of *E. coli* O157:H7 DNA in water using a surface-printed carbon electrode (SPCE) modified with a carbon dot (CD)/ZnO nanorod/PANI composite. The sensor showed high sensitivity with detection at 1.3 × 10^−18^ M compared to other electrochemical DNA sensors due to the enhanced electrical conductivity of its SPCE. Setterington and Alocilja [19] were able to detect as few as 7 CFU of *E. coli* O157:H7 in phosphate-buffered saline (PBS) within 70 min, using an SPCE sensor coupled with immunomagnetic separation (IMS) and biofunctionalized magnetic nanoparticles (MNPs)/PANI. The electrochemical sensor showed a good linear detection range between 10^1^ to 10^5^ CFU/mL in PBS. However, neither a specificity test with mixed bacterial cultures nor testing the sensor’s detection effectiveness in food samples was done to validate the sensor.

Great attention has been focused on ZnO-based nanostructures in the fabrication of biosensors due to their unique optical, piezoelectric and semi-conducting properties and capability to interact with various types of biomolecules [20,21]. Furthermore, ZnO nanostructures have high stability in biological pH values, making them biocompatible and suitable for in vivo applications [20]. However, Zinc-Oxide nanoparticles (ZnO NPs) possess a significant antimicrobial activity by releasing Zn^+^ ions that induce the generation of reactive oxygen species (ROS), making them unsuitable for bacterial cell capturing [21,22]. To overcome this challenge, nanotechnology has offered the advance of fabrication of nanocomposites of metal oxides dispersed in polymeric materials that allow the formation of new chemical bonds to prevent the chance of interference with the bacterial cells [12,21]

In this study, the fabrication and characterization of a simple conductometric immunosensor based on a PANI/ZnO nanocomposite is described for the detection of *E*. *coli* O157:H7. The detection mechanism was based on antibody-antigen binding using a monoclonal anti-*E*. *coli* antibody without the addition of secondary antibodies or any type of labeling reagents. Moreover, the sensor performance was investigated by measuring the relative change in the electrical conductivity of the sensor before and after incubation of the electrode with different concentrations of bacteria.

## 2. Materials and Methods

### 2.1. Materials and Reagents

Chemicals used in this study were analytical grade and were used without further purification. Zinc acetate dihydrate (Zn (CH_3_CO_2_)_2_ 2H_2_O), sodium hydroxide (NaOH), 99.5% aniline, ammonium persulfate (APS), concentrated hydrochloric acid (HCl), bovine serum albumin (BSA), absolute ethanol and methanol were purchased from Sigma-Aldrich, Inc., St. Louis, MO, USA. Glutaraldehyde 25% solution was purchased from BBC Biochemical, Livonia, MI USA. *E. coli* serotype O157 mouse monoclonal antibody (A19Z) was purchased from Invitrogen (ThermoFisher Scientific Inc., Waltham, MA, USA, Cat# MA5-18197).

### 2.2. Preparation of ZnO Nanoparticles

ZnO NPs were prepared by co-precipitation. About 5 g Zn (CH_3_CO_2_)_2_ 2H_2_O (22.8 mmol) was dissolved in 100 mL deionized water in a beaker and stirred for 30 min at 70 °C. In another beaker, 2 g NaOH (50 mmol) was dissolved in 20 mL deionized water and stirred for 10 min. After that, the NaOH solution was added dropwise to the Zn (CH_3_CO_2_)_2_ solution while stirring. The suspension formed was continuously stirred 2 h at 70 °C. Then, the solution was allowed to settle 2–3 h at room temperature and centrifuged at 2000 g for 5 min. The white precipitate was dried in an oven at 150 °C for 2 h and calcinated at 300 °C for 5 h in a muffle furnace. The powder produced was comprised of ZnO NPs.

### 2.3. Preparation of PANI/ZnO Nanocomposite

The PANI/ZnO nanocomposite was prepared by dissolving 0.9 mL (9.7 mmol) aniline monomer in 30 mL 1 M HCl. Subsequently, 0.2 g of the previously prepared ZnO NPs were added to 20 mL deionized water and sonicated for 10 min. The ZnO solution was then added to the aniline solution and continuously stirred for 30 min in an ice bath at 0 °C. After that, 50 mL of 0.25 M APS (12.5 mmol) was added dropwise into the ZnO/aniline solution and continuously stirred for 4 h in an ice bath. The change in solution color from rust brown to dark green indicated the polymerization of the aniline monomer. After that, the solution was passed through a vacuum filtration unit, washed with deionized water, ethanol and methanol, respectively, to remove any impurities. Finally, the dark blue–green powder obtained was dried for 24 h in a vacuum desiccator. The final product was the PANI/ZnO nanocomposite.

### 2.4. Characterization of the Nanostructures

#### 2.4.1. Scanning Electron Microscopy (SEM)

The morphological features of the nanostructures were determined using scanning electron microscopy (SEM) at the Institute of Microstructure Technology (IMT) in Karlsruhe Institute of Technology (KIT) Eggenstein-Leopoldshafen, Germany.

#### 2.4.2. X-ray Diffraction (XRD)

The X-ray powder diffraction patterns of the nanostructures were measured using X-ray diffraction (Rigaku Ultima IV) at the pharmaceutical research center at Jordan University of Science and Technology (JUST) (Al Ramtha, Irbid, Jordan). The XRD unit was equipped with a CuKα radiation source with a wavelength of 0.154 nm over the 2 theta (θ) range of 5.0 to 80.0 degree.

#### 2.4.3. Fourier Transform Infrared Spectroscopy (FTIR)

The quality and purity of the nanostructures were determined using ATR-FTIR spectroscopy (Bruker-Avance) at the Department of Chemistry, Jordan University of Science and Technology (JUST) (Al Ramtha, Irbid, Jordan).

### 2.5. Preparation of Microbial Samples

Three nonpathogenic *E. coli* O157:H7 strains (02:0627, 02:0628, NCTC 12900) used in the current study were obtained from Dr. Rafiq Ahmed, National Microbiology Laboratory, Public Health Agency, Canadian Science Centre for Human and Animal Health, Winnipeg, MB, Canada. Each strain was sub-cultured twice in tryptone soy broth (TSB, Oxoid Ltd., BASINGSTOKE, UK) at 37 °C for 20 h. For tests, 100 μL of the *E. coli* O157:H7 culture was transferred into 10 mL TSB and incubated at 37 °C for 20 h. The number of viable cells was determined using conventional spread plating on tryptone soy agar (TSA, Oxoid Ltd., Basingstoke, UK) in conjunction with serial dilution (1 × 10^1^–1 × 10^7^ CFU/mL) and spectrophotometric analysis (OD_600nm_). *Coronobacter sakazakii* strain 767 was used in the current study as a negative control to characterize the specificity of the sensor developed. *C. sakazakii* was sub-cultured twice in TSB at 37 °C for 20 h. For the experiments, 100 μL of the cultivated *C. sakazakii* was transferred into 10 mL TSB and incubated at 37 °C for 20 h. The number of viable cells was determined using conventional spread plating on TSA and spectrophotometric analysis (OD_600nm_).

### 2.6. Antimicrobial Activity of the Nanostructures

The antimicrobial effect of the nanostructures against three *E. coli* O157:H7 strains was assessed by determining the minimum inhibitory concentration (MIC) using 96 well microtiter plates [23]. A stock solution of 2% (*w/v*) of ZnO NPs and PANI/ZnO nanocomposite were prepared separately by dissolving each nanostructure in sterile deionized water followed by sonication for 10 min. The bacterial cultures were grown overnight, and then the fresh cultures were serially diluted in TSB to obtain a final concentration of 1 × 10^5^ CFU/mL.

The wells of the microtiter plate were filled with 100 µL TSB broth. After that, 100 µL of ZnO NPs or PANI/ZnO nanocomposite stock solution was added to the first well, then six serial dilutions for each nanostructure were made by transferring 100 µL to the next well to obtain concentrations of 20, 10, 5, 2.5, 1.25, 0.625 and 0.3125 mg/mL. Then, 100 µL of the bacterial culture was added to each well. Blank and control wells were included for each bacterial strain. Then, the microtiter plate was analyzed in microplate reader (BioTek ELx800) at a wavelength of 630 nm after 0 and 24 h incubation at 37 °C.

### 2.7. Preparation of Electrode

**Figure 1:**
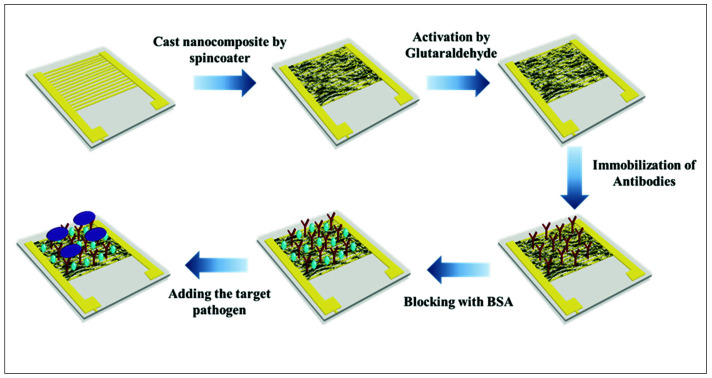
Schematic diagram of sensor’s fabrication.

Prior to modification, the interdigitated gold electrode (size: 1 cm^2^, 1.0 µm gold surface layer, and 12.0 µm copper conductive inner layer) was washed with ethanol and deionized water, in that order, and left to dry at room temperature for few minutes. Then, the electrode surface was coated with 3 layers of PANI/ZnO nanocomposite using a spin coater (Laurell WS-650MZ-23) and dried for few seconds at 70 °C. After drying, 10 µL of 4% glutaraldehyde was dropped onto the modified electrode surface to activate the amine group of PANI and incubated for 1 h at room temperature. Finally, the modified electrode was incubated with 5 µL of 10 mg/mL monoclonal anti-*E. coli* O157:H7 antibodies for 30 min and blocked with 5 µL 5% BSA blocking buffer for 30 min at room temperature. The electrode was washed with PBS at pH 7.0 after each step. For tests, 10 µL of bacterial samples having different concentrations were applied to the sensing site on the electrodes and incubated for 30 min. A schematic diagram of the sensor’s fabrication is illustrated in Figure 1.

The electrochemical characteristics of the modified electrode were measured using a Keithley source meter (model 2425) and I-V software in a current range of 10 nA to 100 µA (forward bias diode) to measure the conductivity, resistivity and relative changes in the sensor’s resistance (ΔR).

### 2.8. Detection in Real Sample

The sensor’s effectiveness for detection in food samples was assessed using ultra-high heat-treated skim milk (UHT) obtained from a local store. Milk samples were spiked with bacterial concentrations from 10^1^ to 10^6^ CFU/mL. After that, 10 µL from each milk sample was applied to the sensing layer on the electrode and incubated for 30 min at room temperature.

## 3. Results and Discussion

### 3.1. Characterization of the Nanostructures

#### 3.1.1. Scanning Electron Microscopy (SEM)

**Figure 2:**
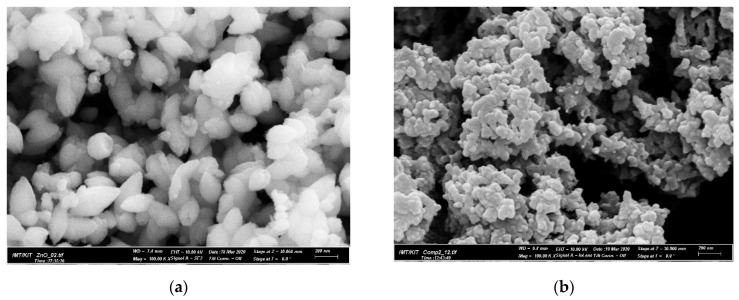
SEM images of ZnO nanoparticles (**a**) and PANI/ZnO nanocomposite (**b**).

The morphological features of the nanomaterials were observed using SEM. Figure 2a shows the SEM images of ZnO nanoparticles with an average size of 100 nm. Nanoparticles with larger sizes observed in the SEM images of the PANI/ZnO nanocomposite in Figure 2b indicate that ZnO NPs were fully coated with PANI, forming ovoidal-shaped particles about 200 nm in diameter.

#### 3.1.2. XRD Analysis

**Figure 3:**
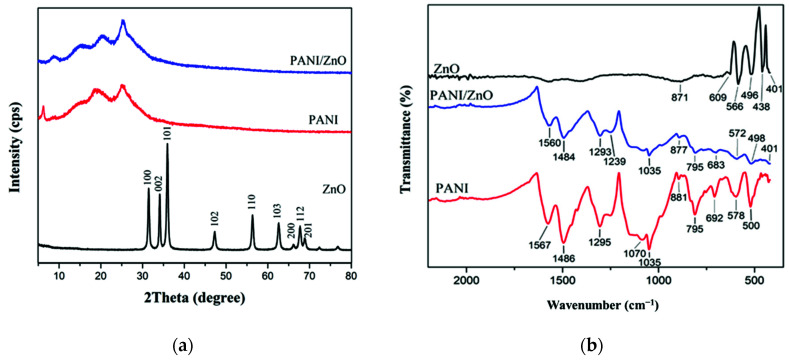
XRD diffraction pattern for ZnO nanoparticles, PANI, and PANI/ZnO nanocomposite (**a**), and FTIR spectra of ZnO nanoparticles, PANI, and PANI/ZnO nanocomposite (**b**).

The ZnO XRD pattern in Figure 3a shows narrow and sharp peaks at 2θ = 31.44, 34.10, 35.94, 47.22, 56.28, 62.54, 66.10, 67.64, and 68.78 degrees. Thereby, these peaks indicate the hexagonal wurtzite structure and the crystallinity of ZnO [24]. A broad and diffused peak is observed in the XRD pattern of PANI around 2θ = 18.8 and 25 degrees, indicating the amorphous nature of PANI [25]. Meanwhile, it can be seen in Figure 3a that the XRD pattern of the PANI/ZnO nanocomposite and PANI are quite similar with a slight shift and higher intensity in the nanocomposite peaks to yield 2θ = 20.4 and 25.56 degrees. This is because of the small amount of ZnO nanoparticles that were below the detection limit of the diffractometer [26].

#### 3.1.3. Fourier-Transform Infrared Spectroscopy (FTIR)

Figure 3b shows the FTIR spectra of ZnO NPs, PANI nanostructure, and PANI/ZnO nanocomposite observed in a wavenumber range between 400–4000 cm^−1^. The PANI spectrum exhibits the main absorption peaks at 1567 cm^−1^ and 1486 cm^−1^, due to the C=N and C=C stretching vibrations of quinoid and benzenoid rings [27]. The absorption intensity ratio between the quinoid and benzenoid rings (1:3) indicates that the polymer is in the conductive form (emeraldine base) [28]. In addition to the 1295 cm^−1^ peak corresponding to the C–N bond stretching in the secondary aromatic amine, the 1070 and 1035 cm^−1^ peaks represent the O=S=O bond stretching of the sulfate anion that serves as a dopant for the polymerization reaction of PANI [25]. The peak at 881 cm^−1^ represents the C–H out-plane bonding in the benzenoid ring. Those at 578 and 500 cm^−1^ represent the characteristic band of PANI the C–N–C bonding mode of the aromatic ring, while the 795 and 692 cm^−1^ bands represent the C–C and C–H stretching of the aromatic ring [26].

The FTIR spectrum of ZnO shows absorption bands located in the lower frequency region between 871–401 cm^−1^, which characterize the Zn–O–Zn and Zn–O stretching [28]. In the PANI/ZnO composite, the characteristic bands of PANI had slightly shifted to 1560, 1484, 1035, and 877 cm^−1^ for the C=N, C=C, SO_2_, and C–H bonds, respectively. Also, the characteristics bands of ZnO were shifted in the PANI/ZnO composite to 795, 683, 572, 498, and 401 cm^−1^ due to the interaction between H–N in PANI and oxygen in ZnO [26]. The occurrence of two bands at 1293 and 1239 cm^−1^ are related to the protonation of C–N in the doped PANI/ZnO composite [29]. The disappearance of the ZnO peak at 871 cm^−1^ indicates the full coverage of ZnO with PANI in the core/shell structure nanocomposite [28].

### 3.2. Minimum Inhibitory Concentration (MIC) of the Nanomaterials

The antimicrobial activity of ZnO NPs, PANI nanostructure, and PANI/ZnO nanocomposite against *E. coli* O157:H7 strains are listed in Table 1. The results indicated that ZnO NPs at a concentration of 0.625 mg/mL significantly inhibited the growth of *E. coli* O157:H7 strains after incubation at 37 °C for 24 h, except for the 02:0627 strain that showed a lower inhibition concentration of 0.3125 mg/mL. Our results were quite consistent with results reported by other studies [30,31,32]. However, the inhibitory effect of ZnO NPs varies depending on the size of the particles used, the number of bacterial cells, and the growth media used in the experiment [33].

In contrast with ZnO-NPs, PANI nanostructure and PANI/ZnO nanocomposite were ineffective against *E. coli* O157:H7 strains as PANI used in this study was in the emeraldine base form and that was consistent with results reported by Kucekova et al. [34]. Further, the negligible effects of the PANI/ZnO nanocomposite against *E. coli* O157:H7 (NCTC 12900) could have been related to the use of hydrochloric acid as a doping reagent in the synthesis of the nanocomposite or the destruction of the bacterial cell wall by electrostatic contact with PANI [35].

### 3.3. Electrical Characterization of the Modified Electrode

**Figure 4:**
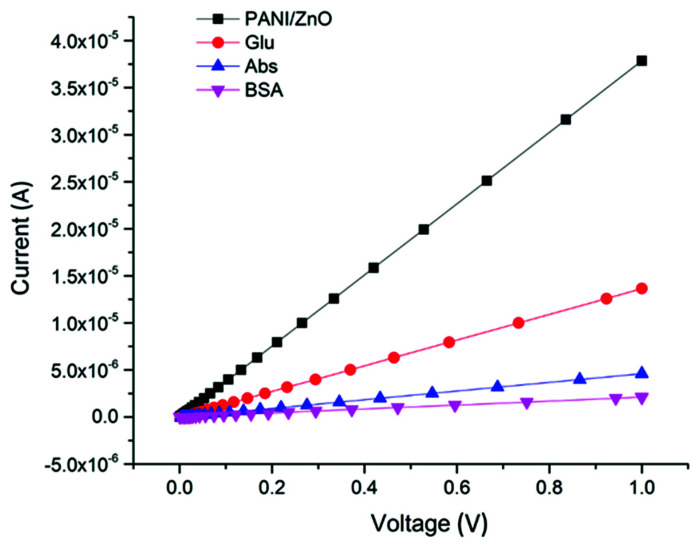
I-V sweep plot for each step of gold electrode modification.

Figure 4 depicts the I-V sweep of the modified gold electrode measured at currents ranging from 10 nA to 100 µA. Good surface contacts between the electrical probes and the interdigitated gold electrode were maintained through the testing process to minimize contact resistance. The PANI/ZnO-coated electrode possessed a relatively high electrical conductivity of 3.78 × 10^−5^ S/m. However, the addition of glutaraldehyde to the sensor hindered the charge transfer process, resulting in a significant decrease in the electrical conductivity to 1.36 × 10^−5^ S/m and consequent increased resistivity [17]. After the antibody immobilization, most of the glutaraldehyde molecules were bound to antibodies, forming large molecular complexes that insulated and occupied the bare regions on the composite film, leading to a further decrease in the sensor’s conductivity to 4.60 × 10^−6^ S/m. The same case emerged for BSA, which blocked the remaining active sites on the electrode surface and lowered the electrical conductivity to 2.12 × 10^−6^ S/m [17,36]. This increase in the electrical resistance of the sensor through the assembly steps indicated a successful binding between the sensor components through fabrication and assembly.

### 3.4. Optimization of Blocking and Incubation Time

To investigate the optimal incubation time for detection using the sensor, 10 µL of *E. coli* O157:H7 at a concentration of 1 × 10^3^ CFU/mL was incubated for 15, 30 or 45 min on different sensors. The relative change in sensor resistance (ΔR) increased with the increase in incubation time from 8.8 kΩ at 15 min to 917.8 kΩ at 30 min, indicating the increase in the strength of the interaction between the antibodies and *E. coli* O157:H7 cells. However, the resistance remained almost the same after 30 min, which indicated that 30 min is the optimum time for irreversible binding between antibodies and the antigen. Hence, if the incubation time were shorter than 30 min, the *E. coli* O157:H7 cells would not be tightly attached to antibodies, leading to their detachment during washing after incubation on the electrode, thus affecting detection.

**Figure 5:**
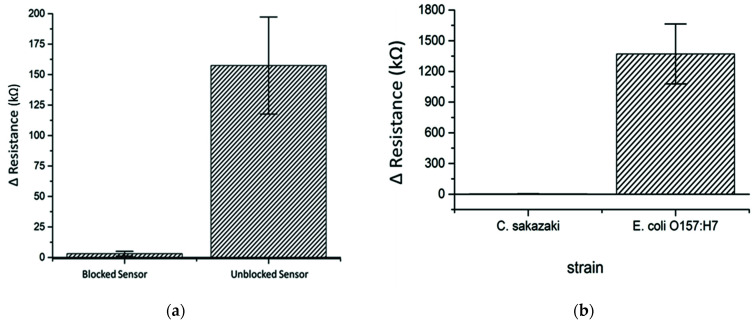
Relative change in sensor resistivity (blocked and unblocked) after incubation with *C. sakazakii* (**a**), and relative change in sensors resistivity of *C. sakazakii* and *E. coli* O157:H7 on *E. coli* O157:H7 nanosensor (**b**).

To determine the possibility of non-specific binding of bacterial cells on the electrode surface, two groups of sensors, which were blocked or unblocked with BSA, were incubated with 1 × 10^3^ CFU/mL of *Cronobacter* *sakazakii* (*C. sakazakii*) as a negative control for 30 min in the presence of anti-*E. coli* O157 antibodies. The results in Figure 5a showed a significant increase in the ΔR from 3.1 kΩ (blocked sensors) to 157.4 kΩ (unblocked sensors) related to increased insulation and interruption in the electrical flow in the electrode caused by the non-specific binding of *C. sakazakii* cells onto the PANI/ZnO nanocomposite film. Therefore, the addition of blocking reagent (BSA in this experiment) is needed to prevent non-specific binding of bacterial cells and bias in measurements.

### 3.5. Specificity and Cross-Reactivity of the Sensors

A cross-reactivity study was done on the antibodies used to evaluate the specificity and selectivity of the immuno-nanosensor. *C. sakazakii* was used as an interference target for anti-*E. coli* O157:H7 antibodies and the response after incubation with the nanosensor for 30 min is depicted in Figure 5b. It can be clearly seen that the *E. coli* O157:H7 nanosensor possessed high sensitivity for *E. coli* O157:H7 compared to *C. sakazakii* using a fixed concentration of 1 × 10^3^ CFU/mL. According to the overall results of ΔR measurements, the anti-*E. coli* O157:H7 antibodies did not have non-specific affinity toward *C. sakazakii,* demonstrating that the sensor was selective and specific for *E. coli* O157:H7 in the presence of another Gram-negative strain.

### 3.6. Calibration Curve of E. coli O157:H7 Sensor

**Figure 6:**
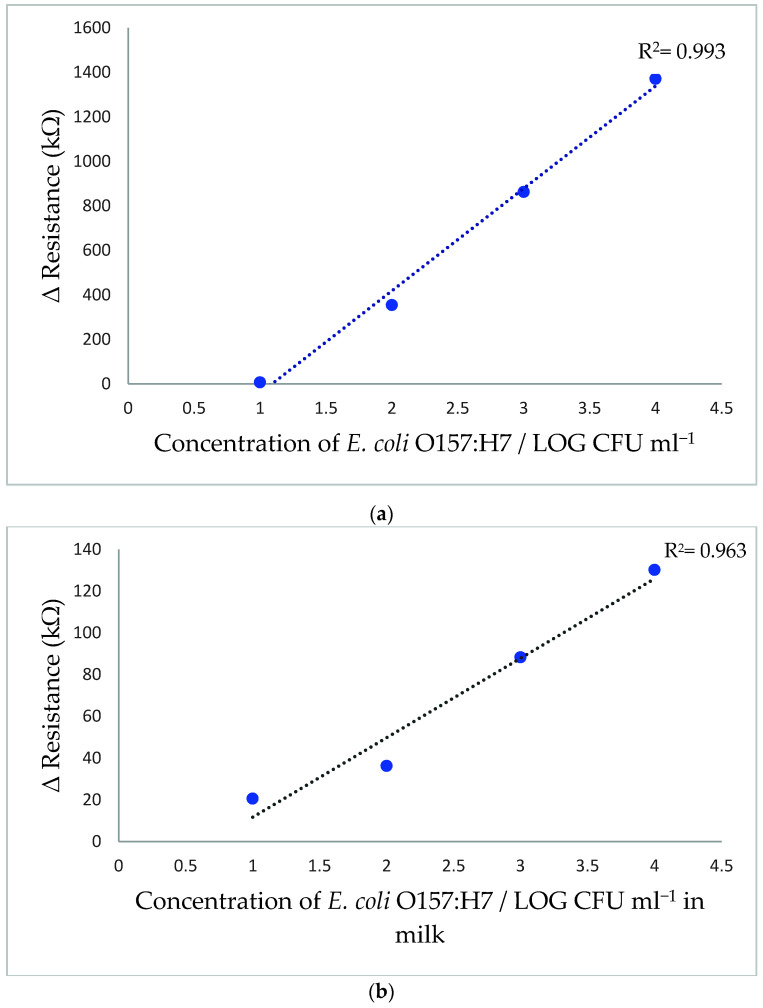
Calibration curve of *E. coli* O157:H7 sensor in 0.1% peptone water (**a**) *E. coli* O157:H7 sensor calibration curve in UHT skim milk (**b**).

The sensor was subjected to the *E. coli* O157:H7 cocktail solution containing strains 02:0627, 02:0628, and NCTC 12900 at concentrations between 1 × 10^1^ to 1 × 10^6^ CFU/mL in 0.1% peptone water. After incubation for 30 min with the sensor, the I-V measurements were taken to calculate ΔR and the corresponding calibration curve was plotted in Figure 6a.

The curve indicated an increase in the sensor resistivity with an increase in *E. coli* O157:H7 concentration. The increase in sensor electrical conductivity can be related to an increase in antigen-antibody immune-complex formation on the electrode surface, which interrupted the electrical flow in the electrode [17,36,37]. The corresponding calibration curve indicated a good linear relationship when bacterial concentration changed from 1 × 10^1^ to 1 × 10^4^ CFU/mL. Correspondingly, the coefficient of determination R^2^ was 0.993 and correlation coefficient *r* was 0.996. The detection limit (LOD) was estimated as 4.8 CFU/mL according to the following equation:

(1) $$LOD=\frac{3\sigma }{S}$$

where *σ* represents the standard deviation of the response and *S* represents the slope of the calibration curve.

On the other hand, the bacterial concentration of 1 × 10^5^ CFU/mL showed a ΔR of 9222.2 kΩ, which was very deviated from linearity. This deviation could be related to the formation of a large amount of antigen-antibody immune-complex on the small sensing area on the electrode surface, which interrupted the electrical flow in the electrode and increased the ΔR, while the increase of bacterial concentrations above 1 × 10^5^ CFU/mL severely disturbed the electrical flow in the electrode, causing a significant fluctuation in electrical response in each time performing the analysis.

The high sensitivity of the detection method can be related to several factors, including the large surface area of the PANI/ZnO nanocomposite that was used to immobilize a large amount of anti-*E. coli* O157:H7 antibodies [38]. In addition, PANI possessed high electrical conductivity that would enhance the electrical path and increase sensor sensitivity [20,39]. Equally important was the use of a low current range from 10 nA to 100 µA for detection, which would increase sensor response to changes in antigen-antibody complex formation on the electrode surface, which would affect the electrical flow and increase resistance. The net effect is an increase in the sensitivity of the sensor.

This study, based on using a novel nanostructure and a highly sensitive PANI/ZnO nanocomposite, achieved an enviable LOD compared with previous immuno-nanosensors developed for detecting *E. coli* O157:H7. For instance, Güner, Çevik, Şenel and Alpsoy [11] used a Chitosan/MWCNT/polypyrrole/AuNPs hybrid platform immune-sensor which had a detection limit of 3 × 10^1^ CFU/mL in PBS. In other work, Wang and Alocilja [40] reported that their magnetic nanoparticles (MNP) and Au NPs reached a detection limit of 1 × 10^1^ CFU/mL in 0.1% peptone water. This low detection limit was believed related to the integration of the immuno-magnetic separation step (IMS) before detection to separate the target antigen and remove any impurities that might interfere with the sensor results [40]. Overall, the detection limit of 4.8 CFU/mL obtained for the sensor developed in the current study is lower than the minimal infective dose of 10 CFU/mL *E. coli* O157:H7, making it applicable for rapid detection of *E. coli* O157:H7 in food.

### 3.7. Application in Milk Sample

The feasibility of the PANI/ZnO nanostructured immunosensor for detecting *E. coli* O157:H7 in spiked UHT skim milk is shown in Figure 6b. The result showed lower ΔR values than those obtained in the calibration curve for *E. coli* O157:H7. This reduction in ΔR values can be related to several factors, such as the precipitation of milk’s mineral salts onto the electrode surface that may facilitate the electrical flow in the electrode. Another factor is the possibility of non-specific binding through hydrophobic or ionic bonds between milk components and the BSA.

Therefore, a new calibration curve (Figure 6b) was developed for *E. coli* O157:H7 detection in UHT skim milk. The sensor showed a linear detection range with an R^2^ = 0.963 at concentrations between 1 × 10^1^ to 1 × 10^4^ CFU/mL and had a good correlation of *r* = 0.981 between measurements. The sensor was able to detect 13.9 CFU/mL in UHT skim milk within 30 min. Furthermore, the same issue observed for bacterial concentration above 1 × 10^4^ CFU/mL in the calibration curve was conducted through the analysis in milk samples.

It must be noted that most of the available commercial techniques require sample pre-enrichment for 24 h before analysis to elevate *E. coli* O157:H7 concentration to a detectable level. However, the sensor developed in the current study responds suitably within 30 min and does not require pre-enrichment. Moreover, the sensor was able to detect *E. coli* O157:H7 in milk without sample dilution prior to analysis, unlike other methods employing immunosensors [41,42]. However, Ranjbar and Shahrokhian [43] developed an Au NP/carbon NP/cellulose nanofiber sensor that showed a significant recovery of between 87.5–113.3% in spiked blood serum samples. This high detection sensitivity could have been related to the use of aptamers instead of antibodies as biorecognition elements for capturing the target bacterium, since they possess high stability and good performance in complex matrices [44].

Overall, the nanostructured immunosensors represent a simple and fast method for analyzing food products to ensure food safety as they possess a suitably low detection limit for E. coli O157:H7 in 0.1% peptone water and UHT skim milk samples compared to other similar techniques. However, the developed sensor has limited application for testing acidic food matrices since the PANI/ZnO nanocomposite electrochemical activity can be adversely affected in solutions with a pH lower than 4 [15].

## 4. Conclusions

In summary, a PANI/ZnO nanocomposite film was prepared and used to develop a nanostructured immunosensor for the detection of *E. coli* O157:H7 based on antibody–antigen interaction. The enhanced electrical conductivity of the PANI/ZnO nanocomposite increased the sensitivity of *E. coli* O157:H7 detection. The sensor showed high selectivity and was used to successfully detect *E. coli* O157:H7 in peptone water and UHT skim milk with a detection limit of 4.8 and 13.9 CFU/mL, respectively, in 30 min. Because of its rapidity and sensitivity, the nanostructured immunosensor described here would appear to have ready application in the food safety and quality control laboratory for the detection of *E. coli* O157:H7.

To the best of our knowledge, this study represents the first use of a PANI/ZnO nanocomposite sensing layer for biosensors to efficiently detect whole bacterial cells, unlike techniques based on detecting bacterial genetic material. However, the new technique has a limitation because the antibodies cannot be regenerated for subsequent detections. Moreover, after a cycle of 5 runs, the interdigitated gold electrode underwent corrosion of the surface or detachment of the gold layer from the substrate. In addition, the repeated washing and drying steps used during the process sometimes led to a loss of the PANI/ZnO nanocomposite film layer, thus preventing the direct transfer of current in the electrode. This problem was observed to occur while in detection mode and yielded “an open circuit”. Investigations are still in progress to improve the electrode materials to enhance the sensitivity and reusability of the nanostructured sensor.

## Figures and Tables

**Table 1:** MIC of ZnO-NPs, PANI nanosheets, and PANI/ZnO nanocomposite against *Escherichia coli* O157:H7.

	Strain Type	MIC (mg/mL) of the Following Nanostructures		
		ZnO-NPs	PANI Nanosheets	PANI/ZnO Nanocomposite
*Escherichia coli* O157:H7	02:0628	0.6250	>20	>20
	02:0627	0.3125	>20	>20
	NCTC 12900	0.6250	>20	10

## Data Availability

Data of this research are available upon request via the corresponding author.
